# Acquired hemophilia A following influenza vaccination: a case report and literature review

**DOI:** 10.1007/s00277-026-07095-w

**Published:** 2026-06-02

**Authors:** Dorit Blickstein, Talia Filipovich-Rimon, Osnat Garach-Jehoshua, Maya Koren-Michowitz

**Affiliations:** 1 Department of Hematology, Shamir Medical Center, Be’er Ya’akov, Israel; 2 Hematology Laboratory, Shamir Medical Center, Be’er Ya’akov, Israel; 3 Gray School of Medicine, Tel Aviv University, Tel Aviv, Israel

**Keywords:** Acquired hemophilia A, Bleeding, Influenza vaccination, Factor VIII inhibitor

## Abstract

Acquired hemophilia A (AHA) is a rare bleeding disorder caused by the development of autoantibodies against coagulation factor VIII. It is idiopathic in approximately 50% of cases, but is also reported in association with malignancies, autoimmune diseases, pregnancy, and medications. We describe an 81-year-old man who developed AHA after receiving an influenza vaccination. The patient was treated with hemostatic therapy and immunosuppressive agents, achieving complete clinical and laboratory remission. Although extremely rare, influenza vaccination may trigger the development of AHA and should be considered in the differential diagnosis of newly diagnosed cases without other identifiable causes.

## Introduction

Acquired hemophilia A (AHA) is a rare autoimmune bleeding disorder caused by the development of autoantibodies (inhibitors) directed against coagulation factor VIII (FVIII), resulting in impaired hemostasis and a tendency toward spontaneous bleeding [1]. The condition is associated with significant morbidity and mortality, particularly among elderly patients with additional comorbidities. The estimated incidence of AHA is 1.3–1.5 cases per million population per year [2]. It predominantly affects older adults, with a median age at onset of 65 years [2]. AHA presents with bleeding and the most common clinical presentation is spontaneous subcutaneous hematoma, observed in about 80% of cases [3]. Other reported bleeding sites include intramuscular (45%), gastrointestinal (21%), genitourinary (9%), and retroperitoneal regions (9%) [3]. Approximately half of AHA cases are idiopathic, while the remainder are associated with autoimmune diseases, malignancies, pregnancy and the postpartum period, as well as infections such as COVID-19. Medications, e.g., penicillin, interferon, and clopidogrel have also been implicated as causing AHA, as well as COVID-19 vaccination [4–7]. Management focuses on two therapeutic goals: (1) control of active bleeding and (2) eradication of FVIII autoantibodies [8]. While multiple reports have described AHA following COVID-19 vaccination, occurrences after influenza vaccination are exceedingly rare [9–11]. We present a case of acquired hemophilia A occurring three weeks after influenza vaccination in an elderly patient.

## Case presentation

Data extraction was approved by the local IRB committee (# ASF-0119-15).

An 81-year-old man was admitted with a painful, extensive hematoma involving the right arm and a prolonged activated partial thromboplastin time (aPTT) discovered on laboratory testing. He had no history of bleeding diathesis, autoimmune disease, trauma, recent surgery, insect bites, use of antiplatelet agents, anticoagulants and non-steroidal anti-inflammatory drugs (NSAIDs), or any new medication. His medical history included type 2 diabetes mellitus (DM), mixed hyperlipidemia, and hypertension. Notably, he had received a quadrivalent influenza vaccine (Fluarix Tetra, Vaxigrip Tetra) three weeks prior to admission. On physical examination, he had a tense, painful hematoma over the right forearm and elbow without neurological deficits. There were no other findings in physical examination. Laboratory findings included mild anemia (Hb 13.3 g/dL; reference 13.5–17.5 g/dL). Platelet (209 K/ul; reference 140–450 K/ul) and leukocyte (7.4 K/ul; reference 4–11 K/ul) counts were normal. Prothrombin time (PT) was normal (12.4 seconds [s]; reference 9.7–13 s; INR 1.02; reference 0.8–1.2), but aPTT was markedly prolonged (95 s; reference 23–36 s, aPTT ratio 3.15; reference 0.77–1.37). A mixing study (1:1 with normal plasma) showed immediate partial correction (aPTT 45 s, aPTT ratio 1.53) but prolongation after two hours incubation at 37 °C (aPTT 77.8 s, aPTT ratio 2.59), consistent with a time- and temperature-dependent inhibitor. Factor VIIl activity was initially 49% and fell to 3.9% after incubation, confirming the presence of a specific FVIII inhibitor. The inhibitor titer was 12.5 Bethesda Units (BU), consistent with a high-titer inhibitor. Von Willebrand factor antigen and activity were normal. Additional workup, including serum electrophoresis, malignancy markers (Prostate Specific Antigen, Cancer Antigen 125, Cancer Antigen 15 − 3, Carbohydrate Antigen 19 − 9, Carcinoembryonic Antigen and Alpha-fetoprotein), lupus anticoagulant, anticardiolipin, and anti–β2-glycoprotein I antibodies, was normal. ANA was positive at a 1:160 titer, while anti-dsDNA was negative, and no further autoimmune evaluation was recommended. Whole-body CT revealed no underlying malignancy.

Treatment was initiated with prednisone 1 mg/kg/day, rituximab 375 mg/m² and tranexamic acid 3 g/day. Within one day, the patient developed a new spontaneous hematoma on the left arm and the Hb level dropped from 13.3 g/dL to 8.2 g/dL. He received a single dose of 50 U/kg of activated prothrombin complex concentrate (aPCC) (16–17); tranexamic acid was discontinued due to safety concerns as it could enhance the prothrombotic effects aPCC through pharmacodynamics synergy [12]. Of note: Emicizumab (a bispecific monoclonal antibody with FVIII-mimetic activity) was not available for AHA treatment at that time. No blood products were required. Coagulation results at diagnosis and during follow up are given in Table 1. Two weeks after the final rituximab dose, the patient achieved a complete clinical and laboratory remission with normalization of FVIII levels and undetectable inhibitor. He subsequently developed severe steroid-induced myopathy, which improved after corticosteroid withdrawal and treatment with pregabalin. During 24 months of follow-up, there was no recurrence of AHA. The patient declined re-exposure to influenza vaccination.

**Table 1:** Laboratory results at diagnosis and during follow up

	Admission	End of Rituximab (day 42)	Late follow up (day 112)
aPTT (seconds/ ratio) N: 23-36/ 0.77-1.25	95 / 3.15	36.6 / 1.21	31.5 / 1.05
Factor VIII level (%) N: 70-140%	3.9	47	128
Factor VIII inhibitor level (Bethesda units) N: <0.6	12.5	0	0

## Discussion

AHA is rare and without prompt recognition and treatment may result in a grim outcome [1, 2]. The mortality rate ranges from 8% to 22%, with the leading causes of death being uncontrolled spontaneous bleeding and infections secondary to immunosuppressive therapy [13]. AHA should be suspected in adult patients who present with spontaneous bleeding, a prolonged aPTT, and no personal or family history of bleeding or anticoagulant use. The pathophysiology of AHA is incompletely understood. It is believed to result from loss of immune tolerance to endogenous FVIII, leading to uncontrolled immune activation against FVIII. Normally, tolerance is maintained through anergy and apoptosis of CD4 + T cells following repeated circulating FVIII exposure. In AHA, this tolerance mechanism fails, leading to uncontrolled immune activation against FVIII. Proposed mechanisms for breakdown of tolerance include [14]:

1. Altered T regulatory (Treg) lymphocyte function, favoring T helper (Th) cell activation.
2. Presence of FVIII-specific Th cells capable of mounting a heightened immune response.
3. Genetic polymorphisms in cytotoxic T lymphocyte antigen 4 (CTLA-4), enhancing Th proliferation and activation.
4. Polymorphisms in the FVIII gene and HLA class II alleles, potentially leading to aberrant antigen recognition and loss of self-tolerance.

Our patient had no personal or family history of autoimmune disease, bleeding, or thromboembolic disorders. He had received annual influenza vaccinations without adverse events since 2013.

The patient had an ANA titer of 1:160, considered a low-to-moderate positive result, indicating the presence of autoantibodies. While such a titer may be associated with autoimmune conditions, it can also occur in chronic infections, certain medications, or even in healthy older adults. We suspect that this ANA positivity may reflect the presence of autoantibodies directed against FVIII.

Cases of AHA following vaccination are extremely rare, although the widespread use of COVID-19 vaccines have led to several reports describing an association with AHA [7]. We could find only 3 previous case reports of AHA following influenza vaccination, their characteristics are summarized in Table 2. All patients received short term immunosuppressive therapy and all achieved complete resolution of clinical and laboratory aberrations.

**Table 2:** Characteristics of influenza vaccine associated AHA cases

Reference	Pirrotta MT et al. [9]	Moulis G. et al. [10]	Chin CS el al. [11]	Current
Age/Gender	66 / F	72 / F	81 / F	81 / M
Comorbidities	NA	Sarcoidosis, hypothyroidism, hypertension, depression	None	type 2 DM, hyperlipidemia, hypertension
Vaccine type	NA	MUTAGRIP	ADIMFLU-S	Vaxigrip Tetra
Onset (days following vaccination)	20	8	17	21
Presenting laboratory				
aPTT (seconds/ratio)	58/3	72/ NA	78.7/ NA	95/ 3/15
Factor VIII (%)	< 1	1	1	3.9
Inhibitor level (BU)	128	8	7	12.5
Treatments	Steroids	Steroids, rituximab	Cyclophosphamide	Steroids, rituximab
Outcome	Resolved	Resolved	Resolved	Resolved
Time to remission (days)	60	120	7	42
Follow-up (months)	NA	NA	NA	24

The cornerstone of AHA management is rapid eradication of inhibitory autoantibodies and control of active bleeding. Current guidelines [15–17] emphasize two parallel treatment modalities, achievement of hemostatic control and eradication of the FVIII inhibitor. Hemostatic agents include recombinant activated factor VII (rFVIla), aPCC, recombinant porcine FVIII (susoctocog alfa), and emicizumab. While rFVIla and aPCC are effective, both carry a potential risk of thrombosis.

Inhibitor eradication is typically achieved with immunosuppression, and most commonly used immunosuppressive agents include corticosteroids, cyclophosphamide, and rituximab. Combination regimens have been associated with higher complete remission (CR) rates (35.2–68.2% with corticosteroid monotherapy; 67-88.5% with combination of corticosteroid and cyclophosphamide; 66-90.9% with combination of corticosteroid and rituximab) [15–18]. Second-line options include mycophenolate mofetil, cyclosporine, tacrolimus, vincristine, and bortezomib. Since AHA primarily affects older adults, prolonged immunosuppressive therapy must be carefully balanced against the risks of infection, viral reactivation (i.e. hepatitis B), and other treatment-related toxicities. After cessation of immunosuppressive therapy, relapse occurs in approximately 25% of patients, with a median time to relapse of 14.7 weeks (IQR 2.9–66.6 weeks) [16].

Inactivated influenza vaccination has been associated with various autoimmune phenomena, including Guillain-Barré syndrome and vasculitis [19]. However, there are limited reports of an association with AHA, as is the case we currently report. According to the Adverse Event Following Immunization (AEFI) system [20], causality at the individual level is difficult to establish; therefore, the relationship is best defined as indeterminate but biologically plausible. Consultation with immunology and infection prevention specialists is recommended prior to future vaccination.

## Conclusion

This case illustrates a possible association between influenza vaccination and acquired hemophilia A in an elderly patient. AHA diagnosis requires high clinical suspicion in patients presenting with unexplained bleeding and prolonged aPTT. A recent vaccination should be included as potential etiologic agent. Further research into the molecular mechanisms underlying loss of FVIII tolerance may enable identification of individuals at risk and inform safer vaccination strategies in susceptible populations.

## Data Availability

No datasets were generated or analysed during the current study.
